# Pachydermodactyly: An Uncommon Benign Digital Disorder

**DOI:** 10.7759/cureus.94382

**Published:** 2025-10-12

**Authors:** Pedro L Almeida, Tiago Félix, Rafaela Evangelista, Pedro M Coelho, Jorge Caldas

**Affiliations:** 1 Physical Medicine and Rehabilitation, Unidade Local de Saúde (ULS) Viseu Dão-Lafões, Viseu, PRT

**Keywords:** digital fibromatosis, msk intervention, pachydermodactyly, pediatric, physical medicine and rehabilitation

## Abstract

Pachydermodactyly is a rare, benign, and non-inflammatory digital fibromatosis, typically presenting in adolescents with painless and progressive swelling of the soft tissues around the proximal interphalangeal (PIP) joints. Most often observed in young males, it is frequently linked to repetitive mechanical behaviors such as finger rubbing, clasping, or habitual trauma. Despite its benign nature and preserved joint function, the condition is often mistaken for inflammatory arthropathies, leading to misdiagnosis and potentially inappropriate management.

We describe the case of a 14-year-old girl with no prior medical history, referred to the Physical and Rehabilitation Medicine from the pediatric department due to a six-month history of finger swelling. The initial concern arose from her family physician, and she denied any pain, trauma, or systemic symptoms. However, she had been taking piano lessons since the age of six. There was no family history of rheumatologic disease. On physical examination, bilateral fusiform swelling of the second and third fingers was observed, centered at the proximal phalanges. The patient had the full range of motion and was pain-free on palpation. Laboratory investigations and radiographs were unremarkable. Given the typical clinical features and absence of inflammatory or structural abnormalities, a diagnosis of pachydermodactyly was made. Treatment consisted of a local infiltration of triamcinolone acetate, which was well tolerated and led to a significant reduction in swelling at follow-up.

We report this case to raise awareness of pachydermodactyly as a rare but important differential diagnosis in adolescents presenting with finger swelling. The condition is frequently underrecognized, particularly in women, and can be mistaken for inflammatory or autoimmune diseases. Early identification can prevent unnecessary investigations and treatments. This case also highlights a positive response to conservative interventional management, supporting individualized care based on clinical presentation and patient preferences.

## Introduction

Pachydermodactyly is a rare and benign form of digital fibromatosis, characterized by progressive, painless soft tissue thickening around the proximal interphalangeal (PIP) joints of the fingers. It most commonly presents in adolescent men, although cases have been reported in women and across a broader age range [1]. The condition is non-inflammatory and non-neoplastic, and despite its harmless nature, it is frequently misdiagnosed as an inflammatory arthropathy such as juvenile idiopathic arthritis or early-onset rheumatoid arthritis [2].

Clinically, the condition presents with symmetric, fusiform swelling over the lateral and dorsal aspects of the PIP joints, commonly sparing the thumbs and fifth digits. Joint function is preserved, and patients typically do not report pain, stiffness, or functional limitations [2].

Due to its rarity and overlapping clinical features with more common disorders, pachydermodactyly is often underrecognized or misinterpreted, leading to unnecessary diagnostic testing and potentially harmful treatments. Early recognition of its hallmark features is essential to avoid misdiagnosis [3,4].

## Case presentation

A 14-year-old right-handed girl with no significant past medical history was referred to the Physical and Rehabilitation Medicine (PM&R) department by the pediatrics department for evaluation of painless swelling in multiple fingers, first noted approximately six months prior to the referral. The initial concern was raised by the family physician due to progressive tumefaction of the digits, without associated pain, redness, or loss of function. The patient reported that the swelling had developed gradually and had not fluctuated significantly since onset. She denied any history of trauma, repetitive strain, or overuse injuries that she could recall. However, upon further questioning, it was noted that she had been taking piano lessons regularly since the age of six, suggesting a possible history of chronic, low-grade mechanical stress involving repetitive finger movements.

There were no associated systemic symptoms such as fever, weight loss, or fatigue. The patient denied any joint stiffness, morning stiffness, or functional impairment. There was no personal or family history of rheumatologic or connective tissue diseases. Her developmental milestones had been achieved appropriately, and she was otherwise healthy and active.

**Figure 1:**
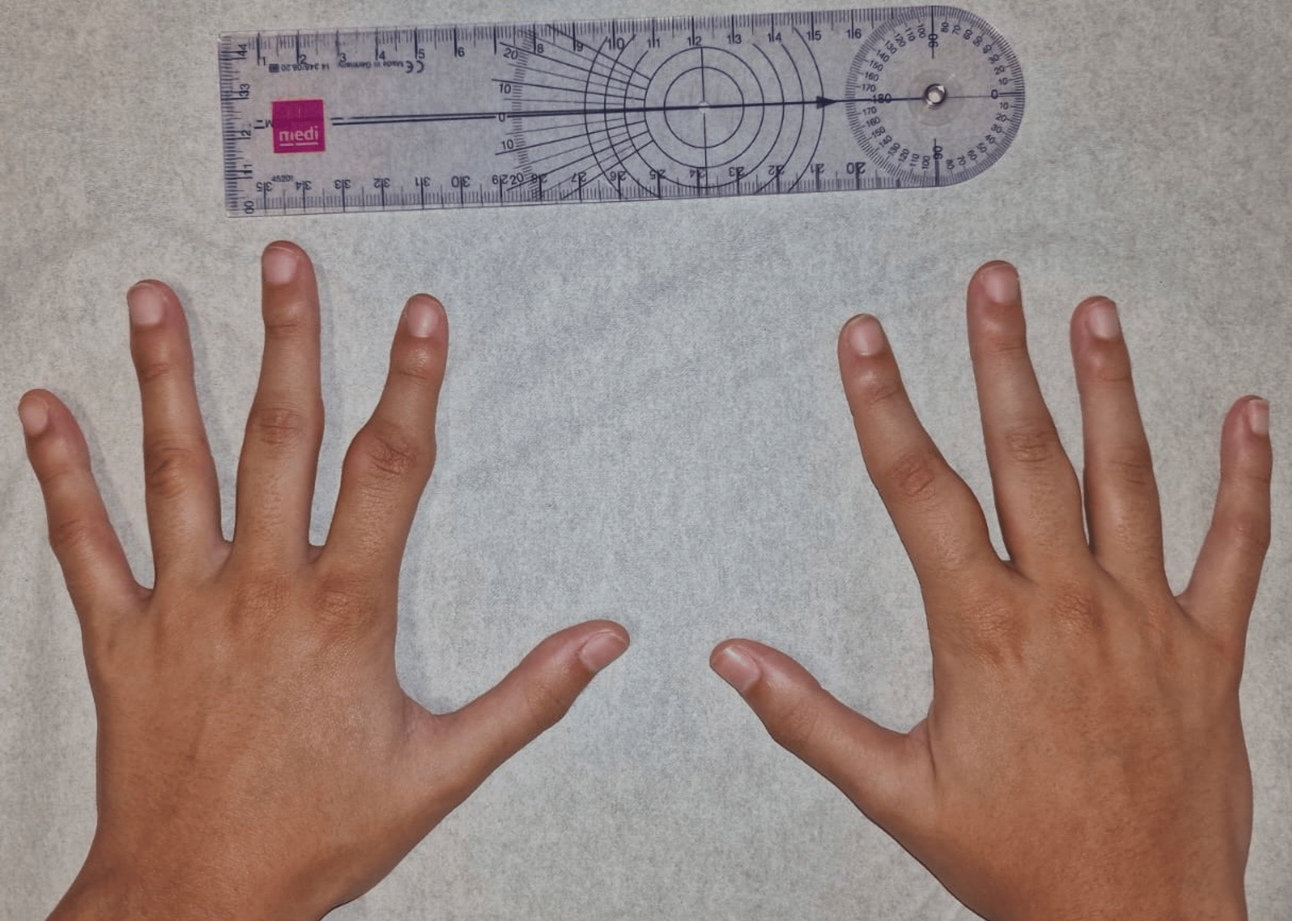
Bilateral symmetric fusiform soft tissue swelling of the second and third fingers, over the proximal phalanges and centered around the proximal interphalangeal (PIP) joints (dorsal view)

**Figure 2:**
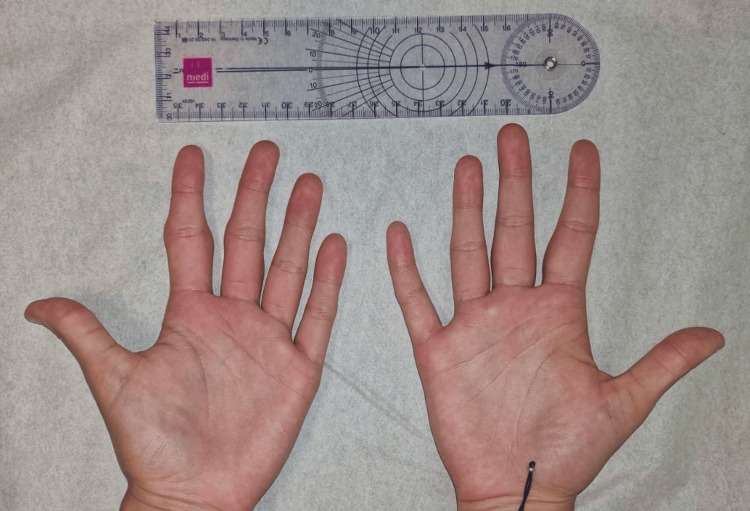
Bilateral symmetric fusiform soft tissue swelling of the second and third fingers, over the proximal phalanges and centered around the proximal interphalangeal (PIP) joints (ventral view)

On physical examination, the patient appeared well and in no distress. Inspection of the hands revealed bilateral, symmetric fusiform soft tissue swelling of the second and third fingers, predominantly over the proximal phalanges and centered around the PIP joints (Figure 1). The skin over the affected areas was intact, with no signs of erythema, warmth, or scaling. There was no evidence of joint effusion, subcutaneous nodules, or nail changes. The swelling was non-tender on palpation, firm in consistency, and fixed to the underlying structures without overlying skin involvement (Figures 1, 2).

The range of motion of all affected fingers was full and painless. There were no deficits in grip strength, dexterity, or fine motor function. No other fingers, including the thumbs and little fingers, were involved, and the wrists, elbows, and other joints were unremarkable.

Laboratory investigations, including complete blood count, erythrocyte sedimentation rate (ESR), C-reactive protein (CRP), rheumatoid factor (RF), and antinuclear antibodies (ANA), were all within normal limits (Table 1).

**Table 1:** Laboratory test results Hb: hemoglobin; RBC: red blood cell; Hct: hematocrit; MCV: mean corpuscular volume; MCHb: mean corpuscular hemoglobin; RDW: red blood cell distribution width; WBC: white blood cell; Neutr: neutrophils; Eosi: eosinophils; Basop: basophils; Lymph: lymphocytes; Mono: monocytes; ESR: erythrocyte sedimentation rate; ALP: alkaline phosphatase; ALT: alanine aminotransferase; AST: aspartate aminotransferase; Creat: creatinine; GGT: gamma-glutamyl transferase; CRP: c-reative protein; ANA: antinuclear antibodies; anti-DsDNA: anti-double stranded DNA antibodies; RF: Rheumatoid factor; HLA: human leukocyte antigen

Laboratory test	Result	Unit	Normal reference range
Hematology			
Hb	13.80	g/dL	12.00-16.00
RBC count	4.91	×10^12^/L	3.85-5.20
Hct	0.43	L/L	0.35-0.46
MCV	87.40	fL	80.00-99.00
MCHb	28.10	pg	26.00-34.00
RDW	13.00	%	11.50-15.00
WBC count	7.41	×10^9^/L	4.00-10.00
Neutr	4.5	×10^9^/L	1.50-8.00
Eosi	0.24	×10^9^/L	0.00-0.50
Basop	0.04	×10^9^/L	0.00-0.30
Lymph	2.15	×10^9^/L	0.80-4.00
Mono	0.48	×10^9^/L	0.00-1.20
Platelet count	289	×10^9^/L	140-440
ESR	12.00	mm/h	0.00-25.00
Biochemistry			
Glucose (fasting)	78	mg/dL	70-110
Urea	29	mg/dL	19-49
Creat	0.69	mg/dL	0.50-1.10
Uric acid	4.30	mg/dL	3.10-7.80
ALT	17	U/L	<34
AST	15	U/L	<49
GGT	10	U/L	<38
ALP	83	U/L	45-129
Sodium	140	mmol/L	132-146
Potassium	4.60	mmol/L	3.50-5.50
CRP	<0.02	mg/dL	<0.50
ANAs	neg	-	-
anti-DsDNA	neg	-	-
RF	<7	UI/mL	<14
HLA-B27	neg	-	-

**Figure 3:**
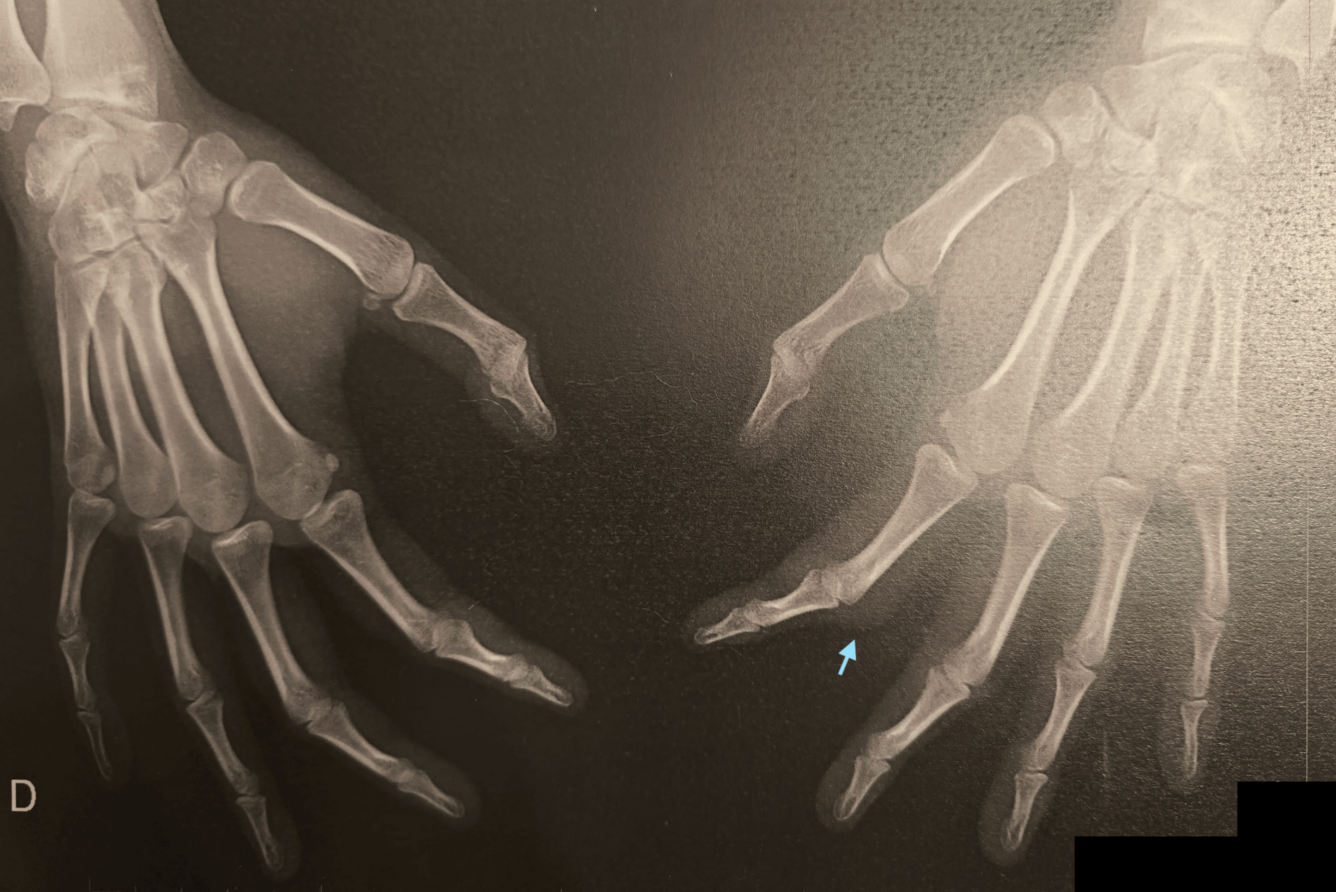
Plain radiographs of both hands with light blue arrow marking an evident soft tissue thickening without bony abnormalities

Plain radiographs of both hands revealed soft tissue thickening around the PIP joints of the affected digits but no bony abnormalities, joint space narrowing, or signs of periarticular erosion (Figure 3).

**Figure 4:**
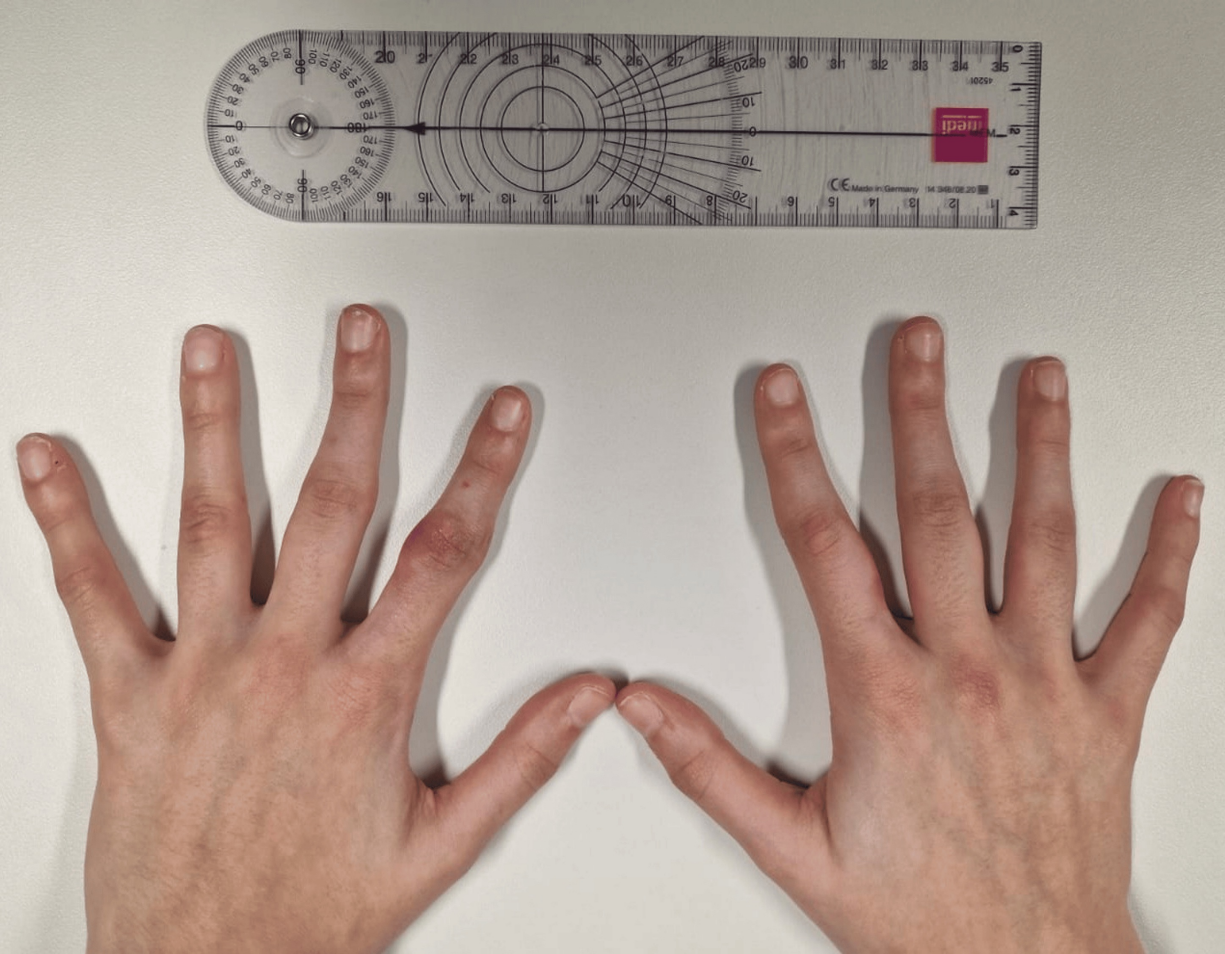
Follow-up eight weeks after the procedure (dorsal view)

**Figure 5:**
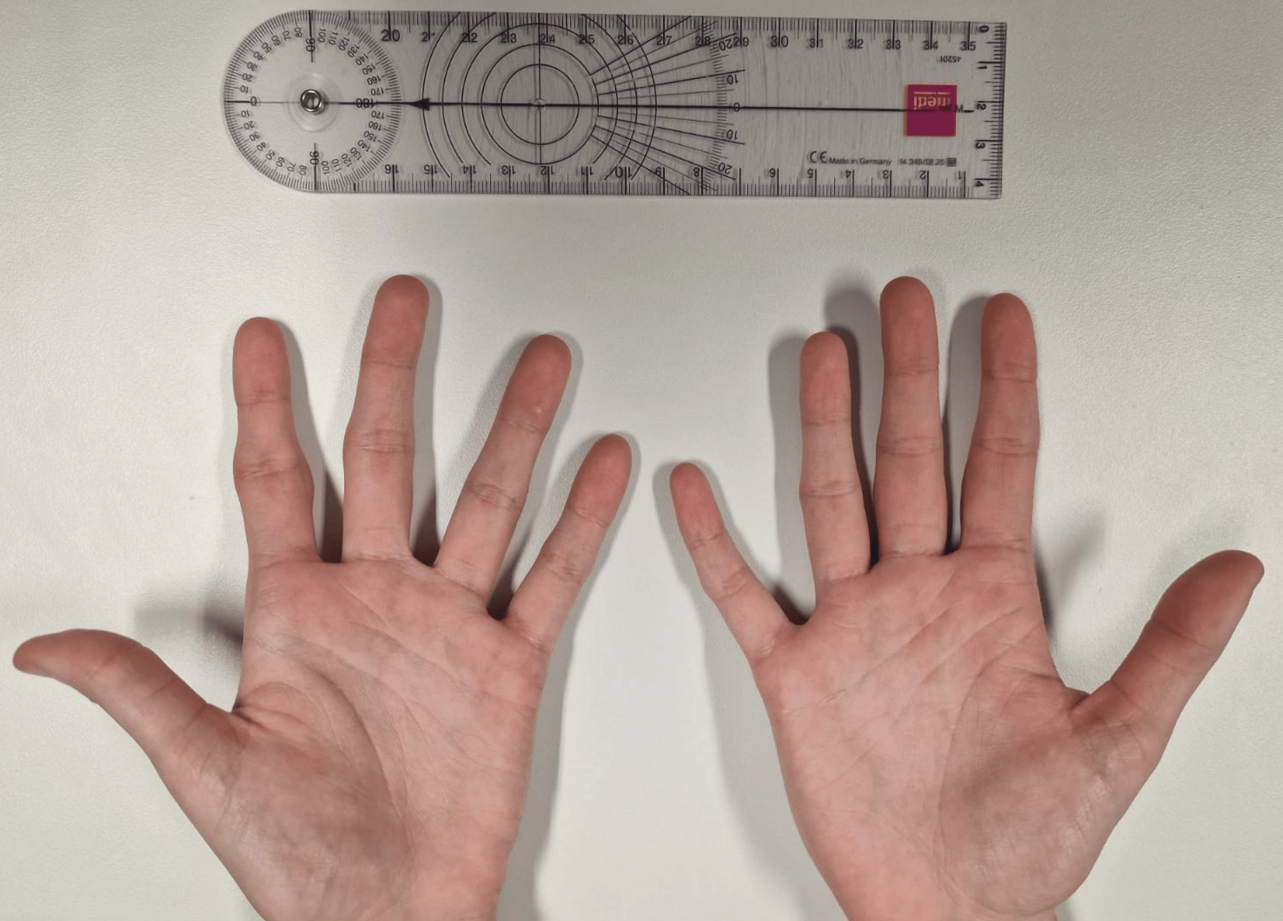
Follow-up eight weeks after the procedure (ventral view)

Given the clinical presentation, absence of inflammatory or autoimmune markers, and imaging findings, a diagnosis of pachydermodactyly was considered. After discussing therapeutic options with the patient and her parents, a conservative interventional approach was adopted. The decision was made to administer an intralesional corticosteroid injection (triamcinolone hexacetonide 20 mg/ml), which was performed in the clinic without complications. At follow-up eight weeks after the procedure, there was a marked reduction in the swelling (Figures 4, 5) with continued absence of pain or functional impairment.

## Discussion

Pachydermodactyly is an uncommon, benign form of digital fibromatosis that poses a diagnostic challenge due to its overlapping features with rheumatologic conditions. Despite its non-inflammatory and asymptomatic nature, its presentation of soft tissue swelling over the PIP joints often leads to misdiagnosis, particularly in young patients. The condition most frequently affects adolescent men with a strong male-to-female predominance. But a number of cases in women, such as the present one, have been reported, demonstrating that gender-based assumptions can lead to diagnostic oversight [1,2].

The etiology remains poorly understood. Several studies suggest an association with repeated mechanical stress or behavior such as finger rubbing, knuckle cracking, or pressure-loading activities involving the hands [3-5]. Although our patient did not report conscious repetitive trauma, she had been attending piano lessons, which may represent a subtle and prolonged mechanical trigger. While playing piano is not traditionally associated with tissue injury, the fine repetitive finger motions over many years may contribute to low-grade dermal stimulation, resulting in fibroblast activation and collagen accumulation - the histological hallmark of pachydermodactyly [6,7].

Histologically, the condition is characterized by thickening of collagen bundles in the reticular dermis, mild fibroblast proliferation, sparse mucin deposition, and an absence of inflammatory infiltrate [4]. These findings strongly support the hypothesis that pachydermodactyly is a reactive fibromatosis rather than an inflammatory or autoimmune condition. Notably, no systemic involvement or internal organ pathology has been linked to the disease, reinforcing its classification as a localized, self-limiting process [5].

From a clinical standpoint, pachydermodactyly typically presents as bilateral, painless, fusiform swelling over the lateral and dorsal aspects of the PIP joints. It most often spares the thumbs and fifth digits and does not impair joint mobility or hand function [2]. These features were consistent with our case, where the patient remained asymptomatic with full range of motion and no signs of inflammation on examination. Laboratory evaluations, including ESR, CRP, rheumatoid factor, and antinuclear antibodies, were within normal limits, and radiographs confirmed soft tissue swelling without joint or bony abnormalities [3].

The differential diagnosis includes a range of rheumatologic, dermatologic, and connective tissue disorders. Juvenile idiopathic arthritis, early-onset rheumatoid arthritis, knuckle pads, systemic sclerosis, and hypertrophic osteoarthropathy must all be considered. Misdiagnosis may lead to inappropriate use of immunosuppressants, corticosteroids or disease-modifying antirheumatic drugs (DMARDs), which carry unnecessary risks in a benign, non-inflammatory condition [6]. Our case emphasizes the diagnostic importance of a thorough clinical evaluation combined with a high index of suspicion for rare but benign entities.

Management of pachydermodactyly is typically conservative, especially in cases without functional impairment or significant cosmetic concern. Patient education and behavioral modification to eliminate contributing mechanical stressors are first-line strategies (6). In more persistent or cosmetically distressing cases, as with our patient, intralesional corticosteroid injections have been reported to produce reduction in swelling possibly by suppressing local fibroblast activity and collagen synthesis [7]. In our case, a single injection of triamcinolone acetate led to a marked regression of the tumefaction at the eight-week follow-up with no reported adverse effects or functional limitations. This outcome suggests that local corticosteroid therapy can be an effective and minimally invasive option for selected patients.

Surgical excision may be considered in refractory or cosmetically severe cases but is rarely necessary and should be reserved for patients who fail conservative or medical therapy [3,4]. Because the disease is non-progressive and lacks systemic consequences, long-term monitoring typically focuses on ensuring diagnostic clarity and avoiding overtreatment.

## Conclusions

This case underscores the value of a comprehensive clinical assessment, which led to an accurate diagnosis and helped avoid unnecessary tests and potentially harmful treatments. Raising awareness and recognizing pachydermodactyly is crucial for ensuring correct diagnosis and appropriate treatment. By documenting this case, we add to the expanding, though still limited, body of literature and emphasize the importance of considering this condition when diagnosing finger swelling in adolescents, regardless of gender or assumed risk factors. Our report highlights the need for clinicians to be alert to unusual presentations, particularly in female adolescents, and stresses the importance of increased awareness of this rare condition in pediatric and primary care settings, where it may often be misdiagnosed. Additionally, the positive response to treatment further supports the role of corticosteroid injections as an adjunct to conservative management in carefully selected patients.
